# Using a Digital Mental Health Intervention for Crisis Support and Mental Health Care Among Children and Adolescents With Self-Injurious Thoughts and Behaviors: Retrospective Study

**DOI:** 10.2196/54816

**Published:** 2024-08-16

**Authors:** Darian Lawrence-Sidebottom, Landry Goodgame Huffman, Aislinn Brenna Beam, Kelsey McAlister, Rachael Guerra, Amit Parikh, Monika Roots, Jennifer Huberty

**Affiliations:** 1 Bend Health, Inc Madison, WI United States; 2 Advocates for Human Potential, Inc Sudbury, MA United States; 3 SleepScore Labs Sleep Solutions, LLC Carlsbad, CA United States; 4 FitMinded Inc, LLC Phoenix, AZ United States; 5 Mental Fitness Clinic Los Angeles, CA United States

**Keywords:** suicide, self-harm, collaborative care, behavioral health, telehealth, telemedicine, eHealth, collaborative, collaboration, suicidal, self-injury, crisis, crises, mental health, self-injurious, anxiety, depression, pediatric, pediatrics, child, children, youth, adolescent, adolescents, teen, teens, teenager, teenagers, mobile phone

## Abstract

**Background:**

Self-injurious thoughts and behaviors (SITBs) are increasing dramatically among children and adolescents. Crisis support is intended to provide immediate mental health care, risk mitigation, and intervention for those experiencing SITBs and acute mental health distress. Digital mental health interventions (DMHIs) have emerged as accessible and effective alternatives to in-person care; however, most do not provide crisis support or ongoing care for children and adolescents with SITBs.

**Objective:**

To inform the development of digital crisis support and mental health care for children and adolescents presenting with SITBs, this study aims to (1) characterize children and adolescents with SITBs who participate in a digital crisis response service, (2) compare anxiety and depressive symptoms of children and adolescents presenting with SITBs versus those without SITBs throughout care, and (3) suggest future steps for the implementation of digital crisis support and mental health care for children and adolescents presenting with SITBs.

**Methods:**

This retrospective study was conducted using data from children and adolescents (aged 1-17 y; N=2161) involved in a pediatric collaborative care DMHI. SITB prevalence was assessed during each live session. For children and adolescents who exhibited SITBs during live sessions, a rapid crisis support team provided evidence-based crisis support services. Assessments were completed approximately once a month to measure anxiety and depressive symptom severity. Demographics, mental health symptoms, and change in the mental health symptoms of children and adolescents presenting with SITBs (group with SITBs) were compared to those of children and adolescents with no SITBs (group without SITBs).

**Results:**

Compared to the group without SITBs (1977/2161, 91.49%), the group with SITBs (184/2161, 8.51%) was mostly made up of adolescents (107/184, 58.2%) and female children and adolescents (118/184, 64.1%). At baseline, compared to the group without SITBs, the group with SITBs had more severe anxiety and depressive symptoms. From before to after mental health care with the DMHI, the 2 groups did not differ in the rate of children and adolescents with anxiety symptom improvement (group with SITBs: 54/70, 77% vs group without SITBs: 367/440, 83.4%; *χ*^2^_1_=1.2; *P*=.32) as well as depressive symptom improvement (group with SITBs: 58/72, 81% vs group without SITBs: 255/313, 81.5%; *χ*^2^_1_=0; *P*=.99). The 2 groups also did not differ in the amount of change in symptom severity during care with the DMHI for anxiety (*t*_80.20_=1.37; *P*=.28) and depressive (*t*_83.75_=–0.08; *P*=.99) symptoms.

**Conclusions:**

This study demonstrates that participation in a collaborative care DMHI is associated with improved mental health outcomes in children and adolescents experiencing SITBs. These results provide preliminary insights for the use of pediatric DMHIs in crisis support and mental health care for children and adolescents presenting with SITBs, thereby addressing the public health issue of acute mental health crisis in children and adolescents.

## Introduction

### Background

Over the past 3 decades, mental health disorders in children and adolescents have increased dramatically in prevalence around the globe, with an estimated 8.8% of children and adolescents having any mental health diagnosis [1]. In the United States, depressive disorders in children have increased from 4% to 6% in 2000 to 13% in 2016 [2]. Anxiety and depression are among the most common mental disorders in children and adolescents [3,4], and there is evidence that these mental health challenges have been exacerbated by the COVID-19 pandemic [5]. Furthermore, rates of self-injurious thoughts and behaviors (SITBs) as well as suicide attempts and completed suicide have been on the rise. Recent estimates report SITBs in 7.5% of children (aged 6-12 y) [6] and 16.9% of adolescents [7]. Indeed, since 2010, there have been alarming increases in pediatric hospital admissions for nonsuicidal self-injury [8-12], with 1 study reporting that hospitalization for suicidal ideation or attempts in children and adolescents more than doubled from 0.66% in 2008 to 1.82% in 2015 [10].

The co-occurrence of recent increases in mental health problems and SITBs in children and adolescents is unsurprising, given that mental health problems, particularly anxiety and depression, may underlie increased risk of SITBs [13]. Indeed, anxiety and depression have been viewed as risk factors for suicidal ideation and attempts [14-16], and those with depression are at particularly high risk for suicide [17]. Therefore, it is of great importance to address mental health issues—particularly anxiety and depression—to prevent SITBs in children and adolescents.

In the United States, the burden of mental health problems and the accompanying risk of suicide and self-harm is exacerbated by an overwhelmed health care system that is increasingly unable to provide adequate mental health care, especially for treatment of acute crises related to SITBs [18]. Most cases of individuals presenting with SITBs are addressed by emergency departments (EDs) that are not specialized in the treatment of SITBs [10,11], and many providers within these EDs report a limited capacity and expertise for treating mental health conditions [19]. ED visit costs have increased by 58% from 2012 to 2019 in the United States [19], reflecting a significant financial burden on both families and the health care system itself. Ultimately, there is a clear and pressing need for accessible, inexpensive, and high-quality mental health care for children and adolescents, especially among those experiencing SITBs [20].

In response to the overburdened mental health care system, digital mental health interventions (DMHIs) facilitated by common digital technologies (ie, internet websites, smartphone apps, or SMS text messages) have emerged as accessible and scalable alternatives to in-person mental health care [21-23]. Evidence suggests that DMHIs that include live interactions, such as video-based coaching and therapy, are similarly efficacious to in-person treatments for common mental and behavioral problems such as anxiety and depression [24-28]. Moreover, DMHIs that use the collaborative care model, in which behavioral care is integrated with primary medical care, are particularly effective for the treatment of mental health problems in children and adolescents [19,29-32].

Despite the demonstrated potential of collaborative care DMHIs and the increasing need for mental health care among children and adolescents presenting with SITBs [33], most commercially available DMHIs exclude those who exhibit such symptoms and may refer them to EDs [34-37]. DMHIs—and particularly those using nonclinical interventions such as behavioral health coaching—often lack the trained personnel necessary for appropriate management of, and follow-up with, those experiencing acute crises related to SITBs. Lack of accommodation for SITBs in pediatric DMHIs produces a cycle of exclusion, referral, and delayed treatment, preventing children and adolescents from receiving the care they need when experiencing acute symptoms.

### Objectives

For mental health treatment to become more accessible to children and adolescents with SITBs, it is crucial that DMHIs offer evidence-based crisis support and ongoing mental health care for children and adolescents at risk of suicide and self-harm [36]. Therefore, this study aims to provide preliminary evidence to inform the further development of digital crisis response services and continuing care for children and adolescents with SITBs and underlying mental health symptoms (ie, anxiety and depressive symptoms). By leveraging 12 months of retrospective data from a pediatric collaborative care DMHI that provides both crisis support and ongoing mental health services to children and adolescents (aged 1-17 y) presenting with SITBs, the purpose of this study is to (1) explore use, mental health symptoms, and demographic qualities of children and adolescents with SITBs who participate in a digital crisis response service; (2) compare anxiety and depressive symptoms of children and adolescents presenting with SITBs versus those without SITBs throughout the duration of care; and (3) suggest future steps for the implementation of digital crisis support and mental health care for children and adolescents presenting with SITBs.

## Methods

### Design and Participants

Children aged 1 to 12 years and adolescents aged 13 to 17 years were eligible for inclusion in this study if they were in care (ie, had a coaching or therapy session) with Bend Health, Inc, between October 1, 2022, and October 1, 2023 (12 months; N=2161). In October 2022, a crisis support protocol was implemented to identify and de-escalate risk in children and adolescents presenting elevated risk of suicide, self-harm without intent to die, harming others, and experiencing violence when not in session with clinical providers (eg, in written communication or in a live coaching session). When a child or adolescent exhibits SITBs in these contexts, a rapid crisis support team (RCST), composed of qualified mental health practitioners experienced and trained in mental health crisis intervention, provide immediate support and work with the child or adolescent and their caregiver or caregivers to develop a safety plan. In this study, those who exhibited risk of suicide or self-harm were included in the *group with* SITBs, and all other children and adolescents were included in the *group without* SITBs; all study participants were in care with Bend Health, Inc.

### Treatment

Bend Health is a collaborative care DMHI for children and adolescents aged 1 to 17 years. All mental health care with Bend Health is delivered using a web-based platform, that is, via live video-based sessions with behavioral health coaches (hereinafter referred to as *coaches*) and licensed therapists (practitioner qualifications are discussed later in this subsection), in a web-based learning resource center, using SMS with mental health practitioners and medication consultation with psychiatric providers (when referred). Most children and adolescents are referred to Bend Health, Inc, by their primary care provider, but they may also enroll through insurance, employer benefits, or direct-to-consumer pathways.

**Figure 1:**
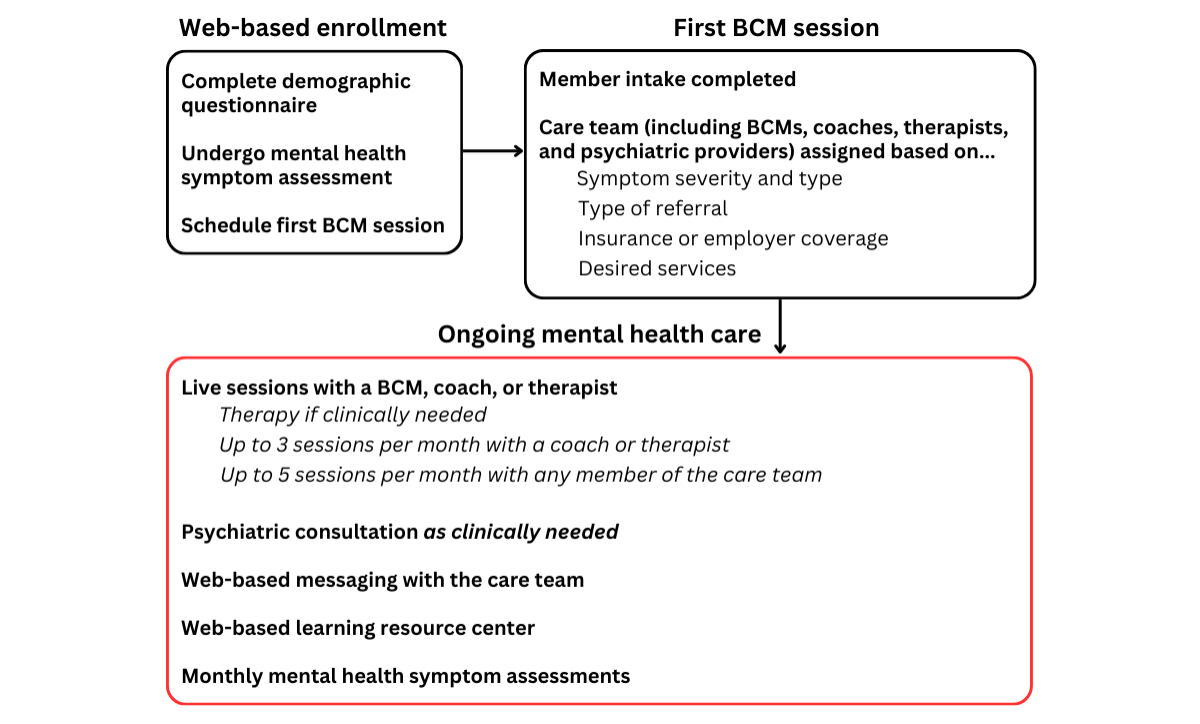
An overview of how members are enrolled in care and receive mental health care at Bend Health, Inc. Activities involved in the following stages of care are identified: enrollment, the first session with a behavioral care manager (BCM), and ongoing mental health care.

Care protocols have been described in detail elsewhere [31,32] and are diagrammed in Figure 1. In brief, caregivers of children and adolescents enroll in care using the web-based Bend Health platform, where they provide demographic information, complete mental health symptom assessments (described in the Measures subsection), and schedule their first session with a behavioral care manager (BCM). BCMs are responsible for coordinating and overseeing a member’s care with Bend Health (ie, they do not deliver mental health care). After a caregiver completes enrollment, the BCM completes member intake and meets with the member and their caregiver in a live video-based session. During this session, the BCM discusses all current mental health concerns; obtains a full history (ie, a history of >2 weeks), including anxiety, depression, and screen for suicidality; and begins planning mental health care. This planning includes the assignment of other Bend Health practitioners to the child’s or adolescent’s care team based on the information gathered. If it is necessary to meet the member’s individual care needs, the BCM will refer the member to 1 of the specialty care tracks at Bend Health, Inc, to provide targeted care.

Coaches are assigned to every child’s or adolescent’s care team. The coach is responsible for leading the delivery of mental health care, and they are required to have one of the following qualifications to work at Bend Health, Inc: (1) be certified by the International Coaching Federation, (2) be certified by the National Board for Health & Wellness Coaching, and (3) hold a master’s degree in a psychology-related field. Other practitioners are assigned to the care team based on the child’s or adolescent’s symptom severity, individual needs, type of referral, insurance coverage, and desired services. Children and adolescents with elevated symptom severity or complex symptom presentation may have a licensed therapist added to the care team. The licensed therapist is responsible for providing a clinical framework to the child’s or adolescent’s mental health treatment, and to work at Bend Health, Inc, they are required to hold a license in one of the following fields: (1) marriage and family therapy, (2) professional counseling, (3) clinical social work, or (4) mental health counseling. If the child’s or adolescent’s referral includes consultation for psychotropic medication, a psychiatric provider (psychiatrist or nurse practitioner) is added to the member’s care team.

Children and adolescents participate in one-on-one live video-based sessions each month with the practitioners on their care team. Coaching and therapy sessions deliver evidence-based behavior change tools to children and adolescents and their families. These sessions aim to facilitate self-reflection and improve self-efficacy and autonomy. Coaches and therapists lead children and adolescents through structured care programs, which were designed by Bend Health, Inc, to target specific symptom domains in a manner that is age appropriate for each member; for example, care programs for children include more simple language compared to care programs for adolescents, and example scenarios are different for these age groups. Caregivers attend live coaching and therapy sessions with their child (aged <13 y). Care for adolescents (aged 13-17 y) is more independent (ie, less focus is placed on the caregiver), and adolescents may attend live coaching and therapy sessions alone. However, their caregiver is required to be nearby (eg, in the same home) during all live interactions with practitioners for safety reasons.

Care program content is also available in a web-based learning resource center, which includes web-based interactive learning tools that reinforce skills learned in sessions to manage and improve symptoms between sessions. The Bend Health care program content is based on the most up-to-date clinical guidelines and practices for pediatric behavioral health, rooted in evidence-based theoretical frameworks such as cognitive behavioral therapy, dialectical behavioral therapy, acceptance and commitment therapy, positive parenting, and mindfulness [38]. Unlimited SMS is also available for caregivers to communicate directly with their child’s care team on a secure portal. Within the same secure portal, caregivers and adolescents are asked to complete mental health symptom assessments (web-based screeners and full validated assessments) at enrollment and approximately once a month thereafter. Mental health symptom assessments are completed between live sessions with practitioners. Children and adolescents may participate in Bend Health care as long as their symptoms persist or until they are discharged from care (eg, by caregiver choice or due to nonengagement).

### Risk Assessment and Intervention Protocol

To provide response and safety planning services for children and adolescents presenting with SITBs and other acute risk behaviors exhibited during nonclinical sessions (ie, sessions with BCMs or coaches) or in asynchronous contact (eg, messages to the care team), Bend Health, Inc, developed a procedure informed by evidence-based practices to identify, intervene with, and manage children and adolescents with SITBs or other mental health crises (eg, intent to harm others) [39,40]. All members of the care team conduct screening for risk at intake and during each live session. To identify suicide risk, practitioners administer the Columbia–Suicide Severity Rating Scale (C-SSRS) [41]. The C-SSRS has been widely used in pediatric populations [42,43] and is validated for use in adolescents aged 13 to 17 years [44,45]. In addition, several studies have used the C-SSRS in children aged 6 to 12 years [46]. The C-SSRS includes items related to wishing to be dead, nonspecific active suicidal thoughts, and plans of suicide (discussed in detail later in this subsection). In addition, practitioners ask children and adolescents about current and past self-injurious behaviors without intent to die, thoughts of hurting another person, and experiencing violence (self or other) in the last 24 hours or being worried about violence in the next 24 hours. Self-harm behaviors are assessed using a 3-item questionnaire. The questionnaire queries whether the child or adolescent has ever harmed or hurt themself (without wanting to die), whether this self-harm happened in the past 2 weeks, and whether the harm was at >1 location *or* required surgical intervention. Children and adolescents are flagged for SITBs when they answer positively to any of the C-SSRS items and indicate engagement in self-injurious behaviors without intent to die in the last 2 weeks.

If a member is identified with SITBs or another risk during intake or any live video-based session with a BCM or coach or if risk is identified in other web-based communication, the BCM, coach, or other Bend Health, Inc, employee (eg, customer service) contacts the RCST. The RCST is a group of mental health professionals qualified to assess and address risk in clinical settings, including those with a master’s degree in a mental health field *and* previous experience managing acute crises in a clinical setting. Upon receiving the notification, an RCST practitioner immediately meets with the member and their caregiver to assess the type and severity of risk, provide crisis support, and develop a safety plan. If a member is flagged for SITBs or other high-risk behaviors during a session with a clinician (therapist or psychiatric provider), the clinician conducts the assessment, provides crisis support, and develops a safety plan with the member and their caregiver.

The risk event assessment involves the evaluation of additional risk factors and protective factors, as well as attempts to identify precipitating circumstances and any plans for suicide or homicide. Specific queried risk factors included previous suicide attempt, depression, access to lethal means, experiencing bullying, social isolation, adverse childhood experience, family history of suicide, and stigma associated with mental health problems or help seeking [47]. Protective factors included limited access to lethal means, connections with friends, family, or community, and positive coping and problem-solving skills [47]. On the basis of the information collected during the risk event assessment, as well as responses to the C-SSRS, the RCST practitioner determines the risk level of the member during the risk event (described in further detail in the Measures section). Then, on the basis of this risk level and the nature of the risk, the RCST practitioner develops a safety plan with the member and their caregiver. The safety plan includes the development of a series of strategies aimed to mitigate the member’s risk (eg, safeguarding the home), as well as the identification of social supports and resources, recognizing warning signs and triggers, self-management skills, and the initiation of immediate actions that the member or caregiver can take to ensure their safety and the safety of others. Safety planning may also involve the alerting (eg, notifying via telephone call) any individuals at risk (eg, intended victims) and the completion of mandatory abuse reporting. In more serious cases, the member’s caregiver may be asked to take the member to the nearest ED, or the RCST practitioner may contact emergency services to go directly to the member’s location.

After the identification of SITBs and other risks, all practitioners in a member’s care team are notified to ensure continuous monitoring and relevant care during sessions. In some cases, BCMs may suggest escalating the child’s or adolescent’s level of care (eg, adding additional therapy sessions). If signs of SITBs or other risks are identified outside of live sessions, an RCST practitioner may offer immediate risk consultation to the care team, or they may contact the caregiver directly to provide immediate crisis support.

### Measures

#### Risk Event Measures

If the responses to C-SSRS item 1, 2, or 6 are *yes*, the member is considered positive for suicide risk. On the C-SSRS, a *yes* response to item 1 or 2 is classified as *mild risk*, a *yes* response to item 3 or 6 (lifetime) is classified as *moderate risk*, and a *yes* response to item 4, 5, or 6 (in the last 3 months) is classified as *high risk*. Data collected before March 1, 2023, were stored in a secure external document. Data collected beginning March 1, 2023 (including the highest suicide risk identified using the C-SSRS), were stored in the electronic health records of Bend Health, Inc.

For the entirety of the study, the following risk event data were collected: random member ID number, date of risk event, type of risk (suicide, self-harm without intent to die, harm to others or homicidal ideation, and abuse or violence), practitioner type (ie, the practitioner who initiated the risk event), and location in care (initial evaluation, follow-up session, or other). From March 1, 2023, onward, these additional metrics were collected: risk factors and protective factors, risk level (mild, moderate, or high), recommended ED evaluation (*yes* or *no*), and calling emergency services on site (*yes* or *no*). The type of risk was identified from responses to the C-SSRS and other practitioner questions. SITB risk was classified using the criteria outlined in Textbox 1 for events flagged with suicide risk, and events flagged with self-harm risk only were always classified as *mild risk*. Identification of risk behaviors could occur at several points throughout participation, including in the first session with a practitioner (labeled “initial evaluation”); after the first session with a practitioner (labeled “follow-up session”); and outside of live sessions, such as via the member chat (labeled “other”).

**Criteria used to determine child and adolescent self-injurious thoughts and behaviors (SITBs) risk based on Columbia–Suicide Severity Rating Scale (C-SSRS) scores and risk factors within the context of a digitally delivered crisis support intervention.**

SITB **risk and criteria**

- Mild: low risk on the C-SSRS; *or* moderate risk on the C-SSRS, and there is a caregiver or other responsible adult present
- Moderate: moderate risk on the C-SSRS, and there is *not* a caregiver or other responsible adult present; *or* high risk on the C-SSRS, and there is a caregiver or other responsible adult present
- High: high risk on the C-SSRS, and there is *not* a caregiver or other responsible adult present

#### Demographic Information

Upon enrollment with Bend Health, Inc, caregivers answer questions about their child’s or adolescent’s demographic information, including date of birth (used to determine age), sex at birth (male, female, or other), gender (boy, girl, transgender, nonbinary, or other), and race or ethnicity (American Indian or Alaska Native, Asian, Black or African American, Hispanic or Latinx, Native Hawaiian or other Pacific Islander, White, or other). As described in further detail in Multimedia Appendix 1, the race or ethnicity response options were expanded starting May 26, 2023, to more clearly align with US census standards. After completing the demographic section, caregivers of children and adolescents complete screening questions for anxiety and depressive symptoms as well as other mental and behavioral health symptoms. These screening questions are repeated approximately every month thereafter throughout the duration of care with Bend Health, Inc.

#### Mental Health Symptoms

Anxiety and depressive symptom severity are assessed for children and adolescents using screeners followed by assessments, which are prompted to be completed on the Bend Health portal monthly. The assessments have been validated for the following age groups [48-52]: children aged 6 to 12 years (caregiver report) and adolescents aged 13 to 17 years (self-report). Different validated assessments were given to caregivers of children versus adolescents (self-report), given best practices for these age groups. Caregivers of children aged 1 to 5 years completed the same assessments as caregivers of children aged 6 to 12 years, but mental health outcome data from children aged 1 to 5 years (161/2161, 7.45%) at baseline were excluded from analysis, given that the measures were not valid for this age group.

For children, caregivers first respond to the anxiety and depressive symptom screening questions derived from the *Diagnostic and Statistical Manual of Mental Disorders, Fifth Edition*, cross-cutting symptom measure for children and adolescents aged 1 to 17 years. These screening questions ask caregivers to report on their child’s symptoms in the past 2 weeks, with responses on a 5-item Likert-type scale ranging from 0=*not at all* to 4=*nearly every day*. There are 2 depressive symptom screener questions and 3 anxiety symptom screener questions. If the response to either depressive symptom screener question or any of the anxiety symptom screener questions is ≥2 (*several days* or more frequently), the caregiver is prompted to complete the full Patient-Reported Outcomes Measurement Information System (PROMIS) questionnaire to assess depression or anxiety, respectively [48,49]. The PROMIS questionnaire has been validated among caregivers of children aged 5 to 17 years [48,49]. The PROMIS depression measure has 11 questions, and the PROMIS anxiety measure has 10 questions. After being prompted with “During the past two (2) weeks, how much (or how often) has your child,” caregivers select the best-fit response to each item using the same 5-item Likert-type scale used in the child screening questions.

For adolescents, the adolescent first responds to the anxiety and depressive symptom screening questions, which consist of the first 2 questions from the Generalized Anxiety Disorder-7 (GAD-7 [50]) and questions derived from the first 2 questions of the Patient Health Questionnaire-9, adolescent version (PHQ-9A [51]). On the basis of their responses to these screening measures, which are referred to as the Generalized Anxiety Disorder-2 (GAD-2) and the Patient Health Questionnaire-2 (PHQ-2), adolescents are prompted to complete the entire GAD-7 and PHQ-9A; both the GAD-2 and the PHQ-2 have been validated as screening tools for identifying probable anxiety and depressive disorders [52,53]. All items in the GAD-2 and PHQ-2, as well as items in the GAD-7 and PHQ-9A, ask adolescents to report on how often they have been bothered by a particular problem (eg, feeling anxious) over the last 2 weeks. Responses are made on a 4-item Likert-type scale ranging from 0=*not at all* to 3=*nearly every day*. If the sum of the scores on the GAD-2 or PHQ-2 is ≥2, the adolescent is prompted to respond to the entire GAD-7 or PHQ-9A, respectively. While the original PHQ-9A includes 9 questions, the version used in this study included 8 questions because the question about suicide and self-harm was omitted.

After the completion of all child or adolescent assessments, assessment scores are saved on a web-based portal for care team review (ie, to guide the care plan), and they are also reported to the caregiver. All demographic information and results from mental health symptom assessments were stored in the electronic health records of Bend Health, Inc, for the duration of the study. For the purposes of this study, mental health symptoms were assessed from January 2023 to October 2023.

### Statistical Analysis

#### Overview

Data were analyzed in the following groups to address study goals: member characteristics and risk event characteristics (aim 1a) and mental health symptom severity (baseline severity: aim 1b; change in severity across participation in the DMHI compared to baseline: aim 2). Throughout, standard descriptive statistics are used to describe group characteristics, namely percentage, mean and SD, and median and IQR. The α level was set to .05 for all analyses. All *P* values were adjusted using the Bonferroni correction to account for multiple comparisons. Data sources were integrated before analysis using deidentified member ID numbers. All analyses were performed retrospectively using R (version 4.2.2; R Foundation for Statistical Computing) [54].

#### Member Characteristics

**Figure 2:**
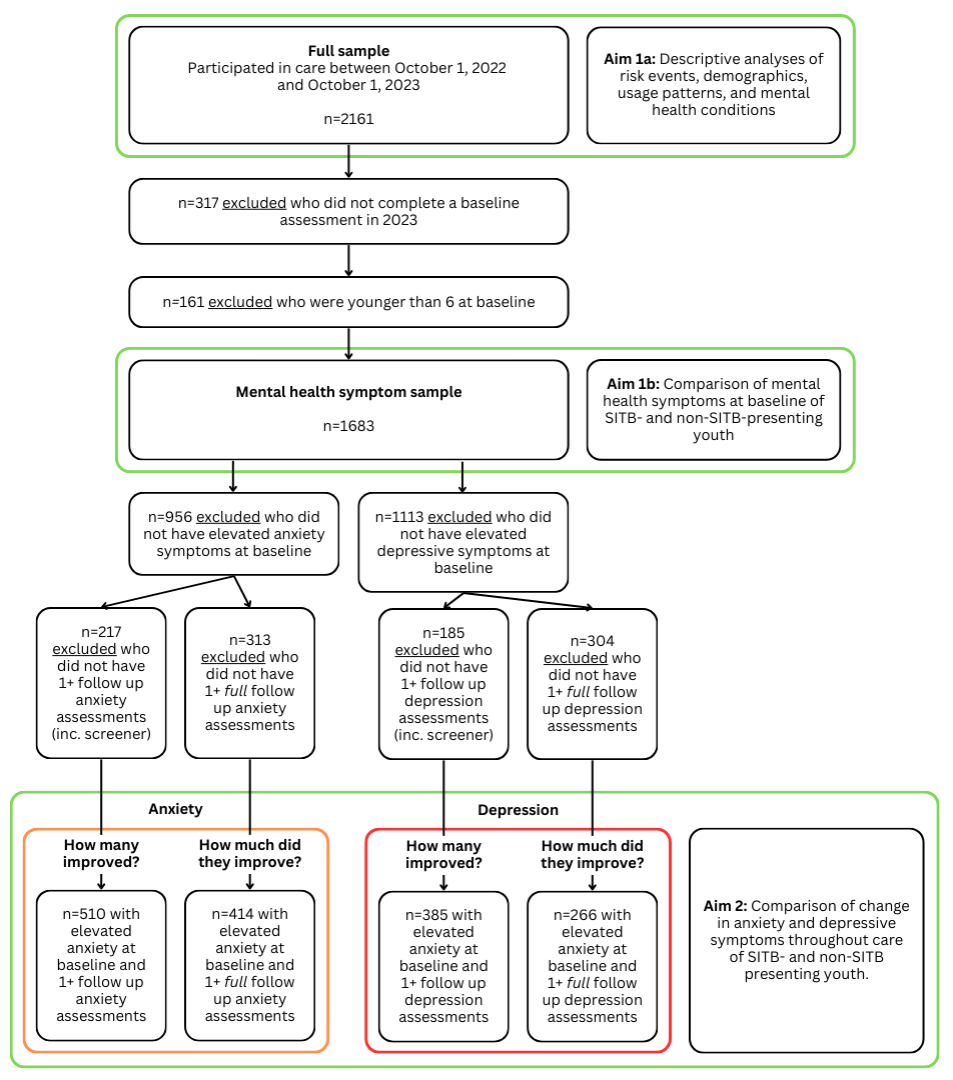
Participant inclusion and exclusion flowchart for each aim of the study. SITB: self-injurious thoughts and behaviors.

As 1 aim of this study was to explore the demographic qualities of members using a crisis intervention, all eligible children and adolescents (aged 1-17 y; N=2161) were included in the descriptive analyses of member demographics, with various exclusion criteria applied for subsequent analyses (the participant flowchart is available in Figure 2). For children and adolescents in the group with SITBs (those who had an SITB event) and the group without SITBs (those with no SITB event), the following characteristics were described: age group (at baseline), sex, gender and sex conformity, race or ethnicity, and mental health conditions reported in the electronic health record (anxiety disorder, depressive disorder, and attention-deficit/hyperactivity disorder). For gender and sex conformity, a reported gender identical to sex at birth was considered *conforming*, and a reported gender different from sex at birth was considered *nonconforming*. Given the many race or ethnicity response options, as well as the changes made to the options partway through the study, the responses were categorized into more general categories (Multimedia Appendix 1).

For participation in behavioral care, the rates of children and adolescents who did not begin coaching or therapy (ie, BCM only) were reported for both groups. The rates of children and adolescents in coaching (coaching only or coaching and therapy) and therapy (therapy only or coaching and therapy) were reported only for children and adolescents who began coaching or therapy. The number of months in care (months from the first session to the last session) were also reported for both groups.

#### Risk Event Characteristics

Member use of crisis support services was quantified for the 12-month period, including the rates of children and adolescents who used the services, the number of total risk events, the types of risk identified, and the number of risk events per member. The timing of the first risk event was identified for all children and adolescents and reported as a percentage of events occurring at intake (first BCM session with Bend Health, Inc) and months in care at the time the first event occurred. Reporting practitioner type and location of event in the member journey was reported for all events. For events recorded after March 1, 2023, the following additional characteristics were reported: SITB risk level, whether ED evaluation was recommended, whether emergency services were called, and whether mandatory reporting was needed. The risk factors and protective factors identified during these sessions were also reported. We did not classify SITBs identified during therapy or psychiatry sessions as a risk event here because the services of the RCST were not required.

#### Mental Health Symptom Severity

Only children and adolescents who had mental health symptom assessments at baseline (ie, the last assessment before the first live session) from January 1, 2023, were included in the analyses of mental health symptom severity (317/2161, 14.67% were excluded). Additionally, only children and adolescents aged 6 to 17 at baseline were included (additional 161/1844, 8.73% were excluded). The rates of elevated (moderate or greater severity) and negative (screened out of completing the *full* assessments) anxiety and depressive symptoms at baseline were reported for each group and compared between the groups using chi-square tests to identify differences in mental health symptom severity.

All subsequent mental health symptom analyses included only children and adolescents with elevated symptoms at baseline (moderate or greater severity; additional 956/1683, 56.8% were excluded from anxiety symptom analyses; additional 1113/1683, 66.13% were excluded from depressive symptom analyses) and at least 1 postcare mental health assessment (after the first BCM, coaching, or therapy session; additional 217/727, 29.9% excluded from anxiety symptom analyses; additional 185/570, 32.5% excluded from depressive symptom analysis) to assess changes in mental health symptoms from before to after care with the DMHI. The rates of symptom improvement from baseline to the last assessment (after the start of care) were calculated as follows: for children and adolescents who did not switch from the child to adolescent assessments during care, a decrease in assessment score *or* screening out of the last assessment was considered symptom improvement; and for children and adolescents who changed from the child to adolescent assessments during care, a decrease in symptom severity (eg, from moderate to mild) was considered symptom improvement. The rates of symptom improvement from the first to the last assessment were reported by group for anxiety and depressive symptoms and then compared between the groups using chi-square tests.

The *change* in mental health symptom severity was quantified for children and adolescents with elevated symptoms at baseline and at least 1 *full* postcare assessment and the same *full* assessment at baseline and after care (ie, no change in assessment type; additional 314/727, 43.2% excluded from anxiety symptom analyses; additional 257/570, 45.1% excluded from depressive symptom analyses). This analysis was performed to ensure that the findings of percentage improvement were not driven by screened-out scores (ie, no *full* assessment). Assessment scores were *z* scored using data from *full* assessments completed at baseline by all children and adolescents enrolled in Bend Health in 2023 (n=1972) to calculate the reference mean and SD (by validated assessment). The *z* scores at baseline were compared between the groups for anxiety and depressive symptoms using Wilcoxon signed rank tests to identify differences at baseline. Next, the change in *z* score from baseline to the last *full* assessment (negative value indicates a reduction in symptom severity) was compared between the groups using 2-tailed *t* tests to determine whether the 2 groups differed in their rates of symptom improvement.

It should be noted that, given individual differences in care duration, the last assessment may have been completed at different times in care for each participant. Thus, the number of months in care from the start of care to the last assessment (ie, screener-only or *full* assessment) and to the last *full* assessment (ie, excluding screener-only assessments) were reported for anxiety and depressive symptoms by group. Wilcoxon signed rank tests were used to assess between-group differences in the timing of assessments. Given the study time frame, it was possible for children and adolescents to have been in care for a minimum of 1 month to up to 12 months.

### Ethical Considerations

Study participants provided informed consent upon enrollment in care with Bend Health, Inc, for primary data collection—a component of regular participation in care—and the use of their data in further analyses. Study participants were not compensated for their participation in the study because data analysis was retrospective. Procedures for this study were approved by the Biomedical Research Alliance of New York (23-12-034-1374; June 5, 2023). All participant data (eg, data from electronic health records) were deidentified before analysis.

## Results

### Member Characteristics

Of the 2161 children and adolescents in the study, 184 (8.51%) had an SITBs risk event (group with SITBs), and 1977 (91.49%) did not have an SITBs risk event (group without SITBs). Comprehensive child or adolescent characteristics for both groups are reported in Table 1. In the group with SITBs, most of the participants (107/184, 58.2%) were adolescents, while 41.9% (77/184) were children. In the group without SITBs, most of the participants (1266/1977, 64.04%) were children, while 35.96% (711/1977) were adolescents. Children and adolescents with SITBs were predominantly female compared to those with no SITBs (118/184, 64.1% vs 986/1977, 49.87%). While the rates of anxiety disorders (group with SITBs: 54/184, 29.4%; group without SITBs: 554/1977, 28.02%) and attention-deficit/hyperactivity disorder (group with SITBs: 21/184, 11.4%; group without SITBs: 305/1977, 15.43%) were similar between the 2 groups, depressive disorders were nearly 5 times more common in the group with SITBs compared to the group without SITBs (33/184, 17.9% vs 74/1977, 3.74%).

**Table 1:** Member characteristics reported for children and adolescents with self-injurious thoughts and behaviors (SITBs) and those without SITBs (N=2161).

Demographics			Group with SITBs (n=184), n (%)		Group without SITBs (n=1977), n (%)
**Age group (y)**					
	Child (1-12)	77 (41.85)		1266 (64.04)	
	Adolescent (13-17)	107 (58.15)		711 (35.96)	
**Sex**					
	Female	118 (64.13)		986 (49.87)	
	Male	63 (34.24)		980 (49.57)	
	Nonbinary	3 (1.63)		11 (0.56)	
**Gender and sex conformity^a^**					
	Conforming	167 (90.76)		1860 (94.08)	
	Nonconforming	17 (9.24)		117 (5.92)	
**Race and ethnicity**					
	Asian	6 (3.26)		97 (4.91)	
	Black or African American	11 (5.98)		107 (5.41)	
	Hispanic or Latinx	11 (5.98)		94 (4.75)	
	White	97 (52.72)		915 (46.28)	
	Other or multiracial	59 (32.07)		764 (38.64)	
**Mental health conditions**					
	Anxiety disorder	54 (29.35)		554 (28.02)	
	Depressive disorder	33 (17.93)		74 (3.74)	
	ADHD^b^	21 (11.41)		305 (15.43)	

^a^A reported gender identical to sex at birth was considered conforming, and a reported gender different from sex at birth was considered nonconforming.

^b^ADHD: attention-deficit/hyperactivity disorder.

Of the 184 children and adolescents in the group with SITBs, 30 (16.3%) did not have coaching or therapy sessions (ie, BCM only), indicating that they had not yet started care. Similarly, 312 (15.78%) of the 1977 children and adolescents without SITBs did not have coaching or therapy sessions. Of the 154 children and adolescents in the group with SITBs with coaching or therapy, 146 (94.81%) were in coaching, and 71 (46.1%) were in therapy. Of the 1665 children and adolescents in the group without SITBs with coaching or therapy, 1608 (96.58%) were in coaching and 389 (23.36%) were in therapy. The group with SITBs had approximately twice the rate of children and adolescents in therapy compared to the group without SITBs (71/154, 46.1% vs 389/1665, 23.36%). For children and adolescents in coaching or therapy, the group with SITBs was in care for a median of 3.63 (IQR 2.20-5.54) months, and the group without SITBs was in care for a median of 3.27 (IQR 1.87-5.17) months.

### Risk Event Characteristics

Of the children and adolescents in the group with SITBs, 84.8% (156/184) had suicidal ideation, and 44% (81/184) had self-harm behaviors, with 28.8% (53/184) flagged as having both suicidal ideation and self-harm. There were 207 SITBs risk events in total, with 89.7% (165/184) of children and adolescents in the group with SITBs having a single event, 8.2% (15/184) having 2 events, and 2.2% (4/184) having 3 events. The first SITB risk events requiring an RCST practitioner took place during the intake process for 44.6% (82/184) of the children and adolescents with SITBs, and the first event occurred a median of 0.03 (IQR 0-0.47; range 0-10.07) months after intake. BCMs initiated 70.5% (146/207) of the SITB risk events, and coaches initiated 18.4% (38/207). Of the 207 SITB risk events, 145 (70.0%) were initiated during an initial evaluation with a BCM or a care provider, and 51 (24.63%) were initiated during a follow-up appointment.

For the 163 events recorded after March 1, 2023, the SITB risk levels were as follows: mild severity=68 (41.7%), moderate severity=75 (46%), high severity=11 (6.8%), and no severity classification=9 (5.52%). ED evaluation was recommended for 7.9% (13/163) of the SITB risk events. No events warranted calling emergency services to the child’s or adolescent’s location, and no events necessitated mandatory reporting. The primary risk factors identified in the SITB risk events were as follows: depression=57.7% (94/163), previous suicide attempt=20.3% (33/163), social isolation=14.1% (23/163), and bullying=12.3% (20/163). The primary protective factors identified were as follows: connections to friends, family, and community=81.6% (133/163), limited access to lethal means=58.3% (95/163), and coping and problem-solving skills=49.7% (81/163). All risk and protective factors identified during SITB risk events are reported in Table S1 in Multimedia Appendix 2.

### Mental Health Symptom Severity

The group with SITBs had a higher rate of elevated anxiety symptoms (94/144, 65.3%) than the group without SITBs (633/1539, 41.13%; *Χ^2^*_1_=30.3, *P*<.001), and they also had a lower rate of negative (ie, screened out) anxiety symptoms (26/144, 18.1% vs 553/1539, 35.93%; *Χ^2^*_1_=17.9, *P*<.001). For depressive symptoms, the group with SITBs had a higher rate of elevated symptoms (101/144, 70.1%) than the group without SITBs (469/1593, 29.44%; *Χ^2^*_1_=90.7, *P*<.001), and they also had a lower rate of negative (ie, screened out) symptoms (31/144, 21.5% vs 876/1539, 56.92%; *Χ^2^*_1_=64.9, *P*<.001). Notably, 51.4% (74/144) of the children and adolescents in the group with SITBs had both elevated anxiety and depressive symptoms, and 21.18% (326/1539) in the group without SITBs had both elevated anxiety and depressive symptoms.

Details regarding the duration between baseline and the last assessment, as well as baseline and the last *full* symptom assessment, are reported in Table S2 in Multimedia Appendix 2. The group with SITBs and the group without SITBs did not differ significantly in their rates of anxiety symptom improvement from baseline to the last assessment (54/70, 77% vs 368/440, 83.6%; *Χ^2^*_1_=1.2, *P*=.32). Similarly, the group with SITBs and the group without SITBs did not differ significantly in their rates of depressive symptom improvement from baseline to the last assessment (58/72, 81% vs 255/313, 81.5%; *χ^2^*_1_=0, *P*=.99). The change in assessment score from baseline to the last *full* symptom assessment did not differ between the groups; baseline symptom severity and the change in symptom severity for both groups, as well as between-group comparisons of these values, are reported in Table 2.

**Table 2:** Baseline and change in symptom severity for children and adolescents with elevated symptoms at baseline and at least 1 full assessment after the start of care and results from the between-group comparisons.

	Group with SITBs^a^, n	Group without SITBs, n	Baseline symptom severity					Change in symptom severity^b^			
			Group with SITBs, *z* score, median (IQR)	Group without SITBs, *z* score, median (IQR)	Comparison^c^		Group with SITBs, Δ *z* score, mean (SD)		Group without SITBs, Δ *z* score, mean (SD)	Comparison^d^	
					*z* score	*P* value				*t* test (*df*)	*P* value
Anxiety symptoms	61	353	0.47 (1.56)	–0.15 (1.53)	–1.94	.09^e^	–1.32 (1.37)		–1.06 (1.31)	1.37 (80.20)	.28
Depressive symptoms	55	211	0.08 (1.62)	–0.22 (1.57)	–2.43	.03^f^	–0.74 (1.36)		–0.76 (1.35)	–0.08 (83.75)	.99

^a^SITBs: self-injurious thoughts and behaviors.

^b^Change from before participating in care with the digital mental health intervention to after participating in care with the digital mental health intervention.

^c^Between-group comparisons of baseline symptom severity (Wilcoxon signed rank tests).

^d^Between-group comparisons of the change in symptom severity (*t* tests).

^e^Statistical (nonsignificant) trend (*P*<.10).

^f^Statistically significant *P* value.

## Discussion

### Principal Findings

We assessed the characteristics and mental health outcomes of children and adolescents presenting with SITBs who participated in a novel digital crisis response service delivered by a pediatric DMHI. Across the 12-month study period, 8.51% (184/2161) of the children and adolescents presented with SITBs and participated in SITB assessment and response services. Children and adolescents presenting with SITBs were older, predominantly female, and had higher rates of elevated anxiety and depressive symptoms than children and adolescents who did not present with SITBs during care. Symptom progression throughout participation did not differ significantly based on SITB presentation, that is, children and adolescents with SITBs exhibited similar rates of symptom improvement over time compared to children and adolescents without SITBs. These findings highlight the critical opportunity to deliver evidence-based crisis response to SITBs in the context of a pediatric DMHI involving nonclinical and clinical care.

The group with SITBs comprised more adolescents than children (107/184, 58.2% vs 77/184, 41.9%). This trend is supported by current national data reporting increased suicidal ideation, self-harm, and death by suicide among adolescents compared to children [55]. Mental health difficulties often increase during adolescence due to the convergence of physiological changes [56], higher levels of stress [57], and relational changes with caregivers and peers [58,59]. Our results highlight the importance of considering age-related developmental differences in the assessment and treatment of SITBs in pediatric DMHIs. However, children engaged in mental health care should still be included in SITB assessments because even mild SITB events in childhood can be a precursor to more serious risk in adolescence or adulthood [60]; for example, 17% of children with suicidal ideation will eventually escalate to attempting suicide [59]. Given our limited ability to investigate within-person development of SITBs over time, longitudinal research is necessary to better understand the long-term impact of DMHIs in mitigating SITBs and associated mental health symptoms in the transition from childhood to adolescence.

Female children and adolescents made up 64.1% (118/184) of the group with SITBs, while female children and adolescents made up 49.87% (986/1977) of the group without SITBs. This apparent sex disparity among those with SITBs is supported by substantial evidence that female adolescents exhibit higher rates of self-harm, suicide attempt, and suicidal ideation across countries [60-63]. In children receiving mental health care, female children were more likely to experience posttraumatic stress disorder [64]. These disparities may have been exacerbated by the stress and social isolation of the COVID-19 pandemic, during which female adolescents reported larger increases in depression and suicidal thoughts than male adolescents [65]. This sex disparity is irrespective of the country of residence: a study that spanned 25 countries highlighted increasing self-harm behaviors in female adolescents since the start of the pandemic [66]. Nonetheless, female children and adolescents are less likely to receive mental health treatment than their male peers [67].

In this study, children and adolescents with SITBs had higher rates of elevated anxiety and depressive symptoms at baseline than those without SITBs (anxiety: 94/144, 65.3% vs 633/1539, 41.13%; depressive symptoms: 101/144, 70.1% vs 469/1539, 30.47%). While other mental health conditions, such as obsessive-compulsive disorder, have been associated with SITBs as well, these conditions are often also comorbid with anxiety and depressive symptoms [68,69]. Indeed, extensive research suggests consistent associations between anxiety and depression and risk of suicide, suicidal ideation, and self-harm in children and adolescents [59,60,62,70]. However, not all children and adolescents with SITBs experience anxiety and depression, and as such, screening for anxiety and depressive symptoms alone does not identify all those with SITBs [71]. In this study, 18.1% (26/144) of the children and adolescents with SITBs tested negative for anxiety symptoms at baseline, and 21.5% (31/144) tested negative for depressive symptoms at baseline. These findings highlight the need for pediatric DMHIs to provide screening and treatment of both SITBs and associated mental health problems.

In this study, the collaborative care DMHI effectively addressed anxiety and depression in individuals with SITBs as well as those without SITBs. This is consistent with prior literature, which has found that therapeutic digital health interventions are effective for treating SITBs. A meta-analysis of SITBs in adolescents across multiple countries found that specific therapies were effective for treating SITBs and self-harm [72], while another meta-analysis found that specific digital health therapies were effective for treating anxiety and depression in children and adolescents from across the globe [73]. However, it is important to note that these meta-analyses revealed that some types of therapeutic interventions had no significant effect on anxiety and depression. The intervention in this study tailors treatment to the child’s or adolescent’s needs and circumstance, which may be 1 reason that participation in the DMHI in this study has been shown to be largely effective for various symptom types and for those with different risk types (eg, SITBs and posttraumatic stress disorder) [31,32,64,74].

Notably, we found that anxiety and depressive symptom severity decreased at similar rates for the group with SITBs and the group without SITBs, with 77% (54/70) of the children and adolescents with SITBs exhibiting improvement in anxiety symptoms after a median of 3.32 (IQR 1.95-4.58) months in care and 81% (58/72) of the children and adolescents with SITBs exhibiting improvement in depressive symptoms after a median of 3.02 (IQR 1.88-3.88) months in care. Although these findings are preliminary, they suggest that SITB presence does not preclude children and adolescents with anxiety and depressive symptoms from benefiting from DMHIs. Several studies have demonstrated that pediatric DMHIs may effectively reduce anxiety and depressive symptoms [31,75,76], with 1 study reporting that reductions in symptoms become clinically significant and persistent for most children and adolescents after 6 coaching sessions [77]. To our knowledge, this study is the first to suggest that children and adolescents with SITBs exhibit similar decreases in anxiety and depressive symptoms to children and adolescents without SITBs while participating in a collaborative care DMHI. These are timely findings, given the increases in suicide and suicidal ideation spurred by the recent COVID-19 pandemic [65,66,75,78] and the consequent need for accessible, scalable, and evidence-based SITBs intervention for children and adolescents. Indeed, few commercially available pediatric DMHIs provide crisis support and mental health services for those exhibiting acute mental health crises or SITBs, citing a lack of adequate risk management strategies, increased liability, and the likelihood of adverse outcomes during care [65]. The findings and methods from this study will ideally inform the further implementation of these services in pediatric DMHIs.

### Strengths and Limitations

The findings presented here should be taken in the context of the strengths of this study. First, this study is novel in that it implements a risk intervention protocol within the framework of a family-centered DMHI. This work is timely, considering the critical need for high-quality and accessible digital crisis support interventions to address the critical public health need of providing quality care to children and adolescents. Second, this study reports on the demographics and mental health symptom outcomes of a group of children and adolescents classified as high risk seeking mental health treatment, thereby adding to the field’s ability to identify and target children and adolescents with SITBs. Third, this study adds to the growing evidence that family-centered, collaborative care DMHIs are well positioned to provide high-quality care to children and adolescents with mental health challenges. Previous studies have demonstrated improvements in a variety of child, adolescent, and caregiver symptoms associated with pediatric mental health care delivered digitally [25,31,32,64,74,76,79]. This is the first study, to our knowledge, to demonstrate improvements in SITBs and comorbid anxiety and depressive symptoms in children and adolescents classified as at risk within the framework of a novel crisis intervention protocol.

Although this study suggests the need for, and the effectiveness of, DMHIs as providers of SITB-related care, it is limited in several aspects. Risk events were only tracked when the RCST was activated, which occurred primarily in the context of sessions with BCMs and coaches. Therefore, our data collection did not adequately gather information from SITBs presented during sessions with a licensed provider (eg, a therapist). Given that many children and adolescents with higher mental health symptom acuity participated in therapy and psychiatry, we expect that there were risk events not captured by this study protocol. However, it should be noted that many of the children and adolescents with SITBs in this study (71/154, 46.1%) participated in therapy during care with the DMHI, but the SITB event was recorded during a coaching or BCM session. Furthermore, 44.6% (82/184) of the risk events occurred during intake, which suggests that SITB risk levels were identified in the early stages of care for many children and adolescents.

The study used a retrospective, pre-post test design. Given the observational nature of retrospective studies, we are unable to make causal inferences; for example, this study did not assess whether mental health problems would have abated in the absence of care with the DMHI. Similarly, it was outside of the scope of this study to investigate associations between variances in care participation (eg, frequency of coaching sessions) and mental health outcomes of children and adolescents presenting with SITBs. Future studies would be strengthened by comparing outcomes among those participating in a DMHI and those in a waitlist-control group or alternative forms of care (eg, in-person care or self-guided DMHIs). In addition, because many children and adolescents had a relatively short duration of participation, we were unable to investigate outcomes associated with long-term participation, particularly in tandem with other demographic factors such as sex and age. However, it should be noted that the study period lasted for 12 months, and thus we were able to summarize outcomes in a relatively large cohort of children and adolescents. Future studies within the Bend Health cohort are planned to assess the effects of longer participation and include follow-up with participants after discharge from the DMHI.

In this study, mental health outcomes were first assessed by screener questions that were followed by full validated assessments only if elevated symptoms were flagged by the screener. With this method, the granularity of our symptom measurement may have been limited, particularly for those with lower symptom severities who may have screened out of completing the full assessments. Of those that completed the full validated assessments (ie, did not screen out) at baseline, 65.85% (727/1104) had elevated symptoms of anxiety and 76.4% (570/746) had elevated symptoms of depression, suggesting that the screener questions alone effectively identified children and adolescents with elevated symptoms. While it is possible that respondents (caregivers or adolescents) may have screened out of completing full assessments due to noncompliance, our primary findings remain consistent across analyses including or excluding screened-out assessments. Some of the assessments included in this study were not validated for all age ranges (eg, C-SSRS was not validated for children and adolescents aged 6-12 y). However, we removed those aged 1 to 5 years where this was applicable. Nonetheless, future studies would be improved by using an assessment protocol that has all study participants complete full symptom assessments and uses validated tools across all age ranges.

In addition, this study is somewhat limited in its assessment of demographic factors. Children aged <6 years were excluded from the analyses of symptom outcomes because the mental health assessments used in this study were not validated for this age range. While less common in this age group, children aged <6 years may experience SITBs [80], and there are mental health symptom assessments designed for use in young children [81,82]. Thus, future studies would be strengthened by the inclusion of measures suitable for children of all ages. The measure of race or ethnicity used for part of this study was limited by a lack of representation racial and ethnic minority people and people of mixed race. Although more than half the cohort (1149/2161, 53.17%) identified as a race other than White, we were unable to assess race or ethnicity as a potential predictor of symptom severity due to the lack of specificity of our measure. In recent years, increases in suicide rates of young people have been particularly pronounced among racial and ethnic minority people [83], and there is evidence that web-based mental health resources may be particularly critical for identifying and mitigating SITBs in populations historically underserved by traditional mental health care [84]. An assessment of the relationship between demographics and SITB-related outcomes was outside of the scope of this study. However, we found that children and adolescents with SITBs were more likely to be female than male, and children and adolescents with SITBs tended to be older than children and adolescents without SITBs. Indeed, it is likely that these demographic factors may predict or otherwise relate to mental health outcomes and responsiveness to behavioral care. Therefore, future research should seek to assess the role of demographic factors in SITB-related outcomes more directly, including the use of a more precise measure of race and ethnicity.

### Future Directions

Considering both current research and the findings presented here, we recommend the following for future research: (1) Conduct qualitative follow-up studies of children and adolescents and their families who have used digitally administered crisis support to increase understanding of the user experience and facilitate consequent improvements to these services. Indeed, qualitative studies have been crucial to expanding and improving digital mental health services in the past [85]. (2) Implement and investigate the effectiveness of web-based intensive outpatient programs for children and adolescents with SITBs and related mental health symptoms. Early evidence suggests that both virtual and in-person intensive outpatient programs for SITBs and related symptoms are acceptable modes of treatment for those who are either transitioning out of, or looking for alternatives to, inpatient programs [86,87]; however, more research is necessary among commercially available pediatric DMHIs. (3) Incorporate peer support and investigate its impact in digital care for SITBs and other acute conditions because a growing body of research suggests that peers, particularly those who have experienced SITBs, may play an important role in SITB prevention and treatment programs [88].

### Conclusions

This study provides preliminary evidence for the utility and effectiveness of SITB assessment, mitigation, and ongoing mental health care in the context of a collaborative care DMHI. Children and adolescents with SITBs exhibited unique demographic characteristics and baseline mental health symptom severity compared to those without SITBs; however, children and adolescents’ symptoms improved throughout care regardless of SITB presentation. Further research is necessary to replicate these results in an experimental setting and determine whether child and adolescent DMHI participation also reduces SITBs and the use of emergency medical services.

## Appendix

Multimedia Appendix 1 Supplemental methods information, detailing the measure of race and ethnicity used in the study.

Multimedia Appendix 2 Supplemental results, including detailed rates of identified risk factors for self-injurious thoughts and behaviors, as well as descriptive statistics and between-group comparisons for the timing of assessments taken after the start of care.

## Abbreviations

BCM: behavioral care manager
C-SSRS: Columbia–Suicide Severity Rating Scale
DMHI: digital mental health intervention
ED: emergency department
GAD-2: Generalized Anxiety Disorder-2
GAD-7: Generalized Anxiety Disorder-7
PHQ-2: Patient Health Questionnaire-2
PHQ-9A: Patient Health Questionnaire-9, adolescent version
PROMIS: Patient-Reported Outcomes Measurement Information System
RCST: rapid crisis support team
SITBs: self-injurious thoughts and behaviors

## Data Availability

The data sets analyzed during this study are not publicly available because this would violate the privacy policy of Bend Health, Inc. However, aggregated and anonymized data that are not associated with individual users and do not include personal information are available from the corresponding author on reasonable request.
