# Supplementary material for: Using a Digital Mental Health Intervention for Crisis Support and Mental Health Care Among Children and Adolescents With Self-Injurious Thoughts and Behaviors: Retrospective Study
Source: JMIR Form Res. 2024 Aug 16;8:e54816. doi: 10.2196/54816 (PMC11364954; doi:10.2196/54816)
Supplement: Multimedia Appendix 1 [file formative_v8i1e54816_app1.doc]

## Methods

The race/ethnicity response options were changed beginning May 26, 2023 to collect more accurate information on member demographics. The updated options were selected based on U.S. census standards. Additionally, starting May 26, 2023, caregivers could select multiple race/ethnicity responses. The original race/ethnicity options were: “American Indian or Alaska Native”, “Asian”, “Black or African American”, “Hispanic or Latino”, “Native Hawaiian or other Pacific Islander”, “White”, and “Other”. On May 26, 2023, the following additional response options were available for selection: “Chinese”, “Vietnamese”, “Native Hawaiian”, “Filipino”, “Korean”, “Japanese”, “Chamorro”, “Other Asian”, “Other Pacific Islander”, “Some other race or multi-racial”, “Mexican, Mexican Am.Chicano”, “Puerto Rican”, “Cuban”. In the present paper, the following categories were used to describe race/ethnicity: White, other or multi-racial, Black/African American, Hispanic/Latino, and Asian. The following responses were categorized as “Asian”: “Chinese”, “Vietnamese”, “Filipino”, “Korean”, “Japanese”, “Chamorro”, “Other Asian”, or “Other Pacific Islander.” The following responses were categorized as “Hispanic or Latino”: “Mexican, Mexican Am., Chicano”, “Puerto Rican”, “Cuban”, “Another Hispanic, Latino, or Spanish origin.” Members with a reported race/ethnicity of “Other” and those that selected multiple race/ethnicity options, were categorized as “Other or multi-racial”.
