# Supplementary material for: Using a Digital Mental Health Intervention for Crisis Support and Mental Health Care Among Children and Adolescents With Self-Injurious Thoughts and Behaviors: Retrospective Study
Source: JMIR Form Res. 2024 Aug 16;8:e54816. doi: 10.2196/54816 (PMC11364954; doi:10.2196/54816)
Supplement: Multimedia Appendix 2 [file formative_v8i1e54816_app2.doc]

## Results

Table S1. Rates of the risk and protective factors identified during SITB events.

| **Risk factor** | **SITB events (n=163),**  n (%) |
| --- | --- |
| Depression | 94 (57.67%) |
| Previous suicide attempt | 33 (20.25%) |
| Social isolation | 23 (14.11%) |
| Bullying | 20 (12.27%) |
| Adverse child event | 15 (9.2%) |
| Stigma of seeking mental health treatment or help | 5 (3.07%) |
| Access to lethal means | 3 (1.84%) |
| Family history of suicide | 2 (1.23%) |
| **Protective factor** | **SITB events (n=163),**  n (%) |
| Connections to friends/family/community | 133 (81.6%) |
| Limited access to lethal means | 95 (58.28%) |
| Coping skills and problem solving | 81 (49.69%) |

Table S2. Months in care when the last and last *full* assessments were completed, and results from the between-group Wilcoxon signed-rank comparisons. Statistically significant P-values (P < .05) are bolded.

| **Symptom** | **Months in care to last assessment**  Median (IQR) | | | **Months in care to last *full* assessment**  Median (IQR) | | |
| --- | --- | --- | --- | --- | --- | --- |
|  | **SITB** | **No SITB** | **Comparison**  *Z* = , *P* = | **SITB** | **No SITB** | **Comparison**  *Z* = , *P* = |
| Anxiety | 3.27 (1.95) | 2.10 (2.77) | *Z* = -2.46, ***P* = .033** | 2.47 (2.97) | 1.97 (2.23) | *Z* = -2.35, ***P* = .038** |
| Depressive | 3.00 (1.95) | 2.40 (2.71) | *Z* = -1.23, *P* = .28 | 2.37 (2.10) | 2.07 (2.33) | *Z* = -1.19, *P* = .29 |
